# PEPCK in cancer cell starvation

**DOI:** 10.18632/oncoscience.252

**Published:** 2015-09-23

**Authors:** Katharina Leithner

**Affiliations:** Division of Pulmonology, Department of Internal Medicine, Medical University of Graz, Graz, Austria

**Keywords:** carcinoma, metabolism, PCK2, phosphoenolpyruvate carboxykinase, starvation

Nutrient and oxygen (O_2_) deprivation are frequent in solid cancers. Angiogenesis is activated early in cancer growth, however, the newly formed vascular network is aberrant and shows irregular blood flow [1]. Moreover, cancer cells themselves contribute to gradients for nutrients like glucose due to high consumption. The concentration of glucose was estimated to be on average 3 to 10 times lower in tumors compared to corresponding normal tissues [2]. Using imaging bioluminescence, glucose levels were shown to be even close to zero in the viable rim around necroses [3]. Cancer cells therefore must adapt to the varying concentration of nutrients, especially glucose. Low availability of glucose dramatically reduces metabolic flux via glycolysis. Accordingly, cancer cells facing chronic glucose limitation were found to increasingly rely on mitochondrial oxidative phosphorylation for sustained proliferation [2]. Besides energy shortage, a reduction of the glycolytic flux potentially results in a drop in cellular intermediates needed for the synthesis of crucial building blocks, like ribose or glycerol - unless these intermediates are generated by alternative metabolic pathways.

**Figure 1:**
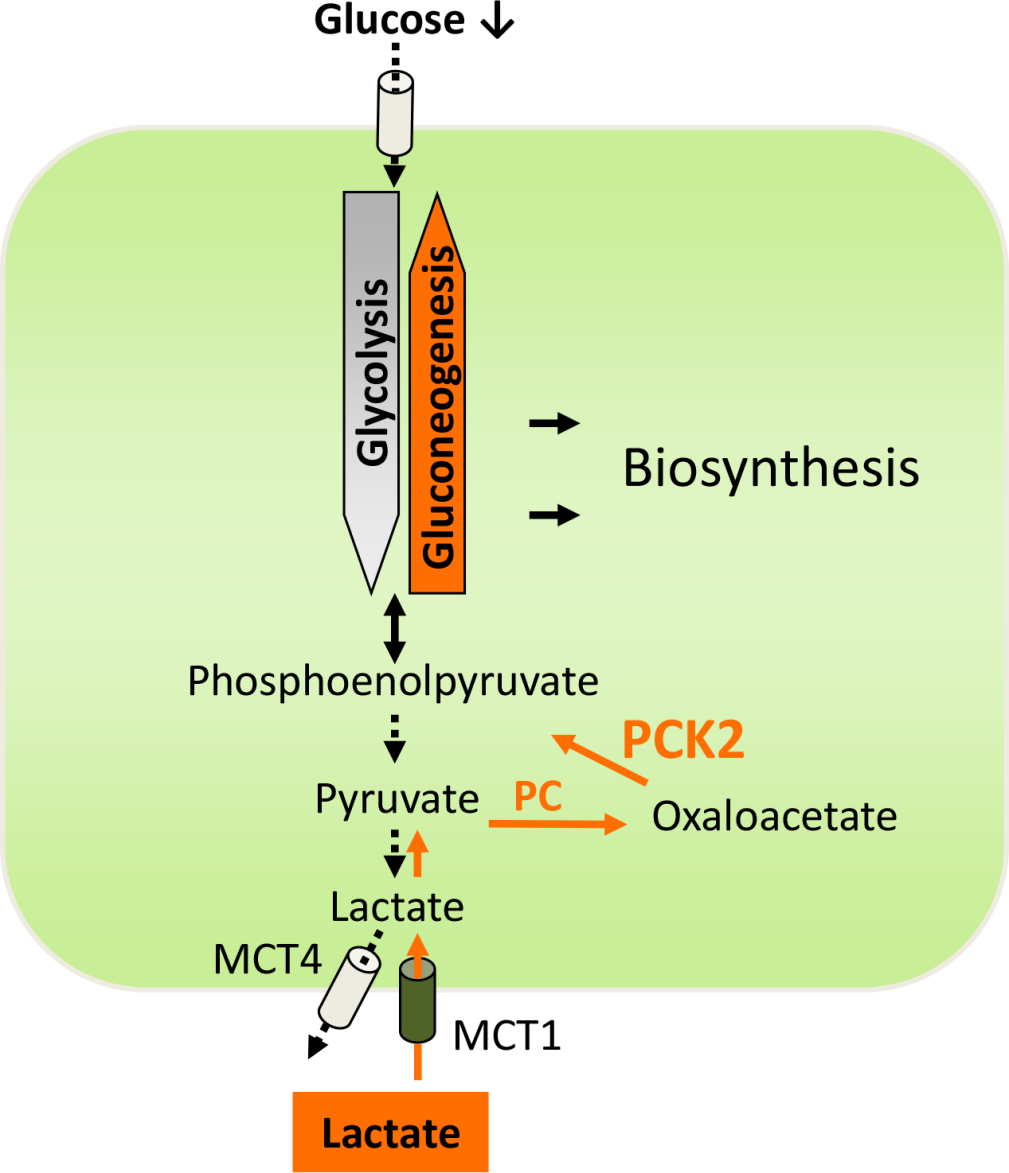
The gluconeogenesis pathway (orange) is an alternative source of phosphoenolpyruvate in lung cancer cells, when glycolysis is reduced under glucose limitation PCK2, mitochondrial isoform of phosphoenolpyruvate carboxykinase (PEPCK); PC, pyruvate carboxylase; MCT1, monocarboxylate transporter 1; MCT4, monocarboxylate transporter 4.

We recently described such a rescue pathway in glucose-deprived lung cancer cells, involving the key gluconeogenesis enzyme phosphoenolpyruvate carboxykinase (PEPCK) [4]. Gluconeogenesis results in the generation of glucose from smaller carbon substrates such as lactate (Fig. 1) or amino acids. In many aspects gluconeogenesis is the reverse reaction of glycolysis, but differs in the requirement for PEPCK and fructose-1,6-bisphosphatase (FBP). PEPCK, the key enzyme of gluconeogenesis, converts oxaloacetate to phosphoenolpyruvate (PEP). Two isoforms of PEPCK exist, a cytoplasmic isoform (PCK1, PEPCK-C) and a mitochondrial isoform (PCK2, PEPCK-M). Besides gluconeogenesis, PEPCK also plays a key role in “abbreviated” gluconeogenesis pathways, e.g. in the generation of glycerol (glyceroneogenesis). In our study [4] we found that PEPCK (the mitochondrial isoform PCK2) is expressed in lung cancer tissue and lung cancer cells. PCK2 expression and activity were enhanced under low glucose conditions. In isotopic tracer experiments we found that lactate is converted via pyruvate carboxylase and PEPCK (PCK2) and thereby contributes to the pool of PEP in lung cancer cells cultured under low glucose (Figure 1). Inhibition and knockdown of PCK2 enhanced apoptosis and cell death under glucose deprivation in lung cancer cells with inactivating mutations of the tumor suppressor liver kinase B1 (LKB1), but not in a lung cancer cell line with wild-type LKB1 [4]. Moreover, inhibition of PEPCK reduced the growth of 3D-lung cancer cell spheroids [4]. Similar findings on a pro-survival role of PCK2 under low nutrient stress were obtained in breast and colon carcinoma cells and published subsequently [5]. In line with a cancer-promoting role of PEPCK, Zhang et al. [6] recently reported that the tumor suppressor p53 down-regulates PCK1.

The hostile cancer microenvironment is increasingly considered to play role in carcinogenesis, and adaptation to these conditions creates metabolic vulnerabilities in cancer cells [7]. PEPCK (PCK2) has a unique position in cell metabolism by acting as a bottle neck in biosynthetic pathways starting from alternative carbon sources, like lactate (Fig. 1). The mentioned studies including our study, suggest, that PEPCK plays an important role in the adaptation of cancer cells to glucose deprivation. However, further research is warranted to clarify the role of PEPCK (PCK1 or PCK2) in cancer cell metabolism. First, the major metabolic downstream pathway fed by PCK2 is at present unknown. Numerous potential metabolic downstream pathways for PEPCK derived PEP exist, e.g. conversion to serine, conversion to glycerol for lipid synthesis, utilization by the non-oxidative branch of the pentose phosphate pathway to generate ribose- 5-phosphate, or synthesis of glucose-6-phosphate, which may be stored in cancer cells in the form of glycogen. The fate of PEPCK-derived PEP, however, might depend on the actual cellular requirements and the metabolic microenvironment. Second, it is not known, how the capacity to utilize the PEPCK pathway is influenced by the tissue of origin on the one hand, and by oncogenes and tumor suppressor genes on the other hand. Interestingly, in renal cancer the gluconeogenic enzyme FBP1, which acts downstream of PEPCK, was found to inhibit tumor progression. Thus, tumor-type specific differences in the expression/activation of either PCK1 or PCK2 might exist. Third, in contrast to hypoxia, only little is known about the spatial and temporal variation of glucose availability in different cancers. Furthermore, the overall contribution of glucose-deprived cancer cell populations to tumor growth and metastasis has yet to be determined.

The finding that activation of the gluconeogenesis pathway, previously thought to be restricted to specialized cells in the body (liver, kidney, intestine and adipocytes) occurs in lung cancer cells, revealed an unexpected metabolic flexibility of cancer cells. To target these or other stress-induced metabolic pathways might potentially kill cancer cells within their hostile microenvironment. However, as with any potential treatment targeting cancer metabolism, the side-effects of manipulating PEPCK or other central metabolic routes have to be considered carefully.
